# Development and Characterization of *Triticum aestivum*-*Aegilops longissima* 6S^l^ Recombinants Harboring a Novel Powdery Mildew Resistance Gene *Pm6Sl*

**DOI:** 10.3389/fpls.2022.918508

**Published:** 2022-06-02

**Authors:** Xiubin Tian, Qifan Chen, Chao Ma, Wenqiang Men, Qianqian Liu, Yue Zhao, Jiajun Qian, Ziwei Fan, Jingnan Miao, Jinqiu He, Sunish K. Sehgal, Huanhuan Li, Wenxuan Liu

**Affiliations:** ^1^State Key Laboratory of Wheat and Maize Crop Science, Henan Agricultural University, Zhengzhou, China; ^2^College of Life Sciences, Henan Agricultural University, Zhengzhou, China; ^3^Department of Agronomy, Horticulture and Plant Science, South Dakota State University, Brookings, SD, United States

**Keywords:** common wheat, *Ae. longissima*, 6S^l^#3 recombinants, powdery mildew resistance, physical mapping

## Abstract

Powdery mildew of wheat is a foliar disease that is spread worldwide. Cultivation of resistant varieties is the most effective, economical, and environmentally friendly strategy to curb this disease. Powdery mildew resistance genes (*Pm*) are the primary resources for resistance breeding, and new *Pm* genes are in constant demand. Previously, we identified *Aegilops longissima* chromosome 6S^l^#3 as a carrier of powdery mildew resistance and designated the resistance gene as *Pm6Sl*. Here, we reported the design of 24 markers specific to 6S^l^#3 on the basis of the full-length cDNA sequences of 6S^l^#3 donor *Ae*. *longissma* accession TA1910, and the development of wheat-*Ae. longissima* 6S^l^#3 introgression stocks by *ph1b-*induced homoeologous recombination. Further, 6S^l^#3 introgression lines were identified and characterized by integration analysis of powdery mildew responses, *in situ* hybridization, and molecular markers and *Pm6Sl* was mapped to a distal interval of 42.80 Mb between markers Ael58410 and Ael57699 in the long arm of 6S^l^#3. Two resistant recombinants, R43 (T6BS.6BL-6S^l^#3L) and T27 (Ti6AS.6AL-6S^l^#3L-6AL), contained segments harboring *Pm6Sl* with less than 8% of 6S^l^#3 genomic length, and two markers were diagnostic for *Pm6Sl*. This study broadened powdery mildew resistance gene resources for wheat improvement and provided a fundamental basis for fine mapping and cloning of *Pm6Sl* to further understand its molecular mechanism of disease resistance.

## Introduction

Bread wheat (*Triticum aestivum* L., 2*n* = 6*x* = 42; AABBDD) is a hexaploid species of the genus *Triticum* in the grass family *Poaceae* (or *Gramineae*). It is the most extensively grown and traded cereal crop with the highest monetary value and the second most-produced cereal after maize. Therefore, the sustainable production of wheat is essential for social progress and stability worldwide. Powdery mildew caused by the fungus *Blumeria graminis* f. sp. *tritici* (*Bgt*) is one of the most damaging foliage diseases of wheat. Severe infection can cause yield loss of 5–30% and decrease grain quality in epidemic years (Fried et al., 1981; Bennett, 1984; Conner et al., 2003; Morgounov et al., 2012). The development and cultivation of resistant varieties is currently the most effective, economical, and environmentally friendly approach to constraining yield and grain quality losses caused by this disease (Wang et al., 2005). As resources for resistance breeding, many powdery mildew resistance genes (*Pm*) have been identified and introgressed into contemporary wheat varieties from landraces, ancestral species, and other wild relatives. Currently, at least 100 permanently and temporarily designated genes for powdery mildew resistance have been documented (McIntosh et al., 2017; Li et al., 2020; Zhang et al., 2020; He et al., 2021). Fourteen cataloged *Pm* genes have been cloned, including *Pm1, Pm2, Pm3, Pm4b, Pm5e, Pm8, Pm17, Pm21, Pm24, Pm38, Pm40, Pm41, Pm46*, and *Pm60* (Brunner et al., 2010; Hewitt et al., 2021; Sánchez-Martín et al., 2021; Yang et al., 2021). Only a fraction of the known *Pm* genes, such as *Pm2, Pm4b, Pm8, Pm13*, and *Pm21*, have been widely deployed in wheat production (Yang et al., 2013, 2021; Xing et al., 2018). A large number of *Pm* genes have been defeated by virulent races, and others could not be deployed in wheat breeding owing to either race-specificity or deleterious gene linkage drag (Klindworth et al., 2013; Tan et al., 2019). Consequently, new *Pm* genes for continuous improvement of wheat are still in demand.

Cultivated bread wheat has a large genome of 17 giga-base pairs (Gb) comprising three subgenomes, A, B, and D, which were contributed by *T. urartu* Tumanian ex Gandilyan (2*n* = 2*x* = 14, AA), *Ae. speltoides* Tausch (2*n* = 2*x* = 14, SS), and *Ae. tauschii* Coss. (2*n* = 2*x* = 14, DD) (Dubcovsky and Dvorak, 2007; El Baidouri et al., 2017). Wild species of common wheat serve as important gene resources for wheat improvement. Many of these species contain chromosomes that are not homologous to those of wheat, and the genes they bear are difficult to transfer to stable wheat lines. Since those chromosomes are functionally similar or homoeologous to wheat chromosomes, they can often be induced to pair and recombine with wheat chromosomes by suppression of strict bivalent formation, which is controlled by genes *Ph1* on chromosome arm 5BL and *Ph2* in homoeologous group 3 chromosomes (Riley et al., 1968; Sears, 1977; Qi et al., 2007). Other methods of achieving chromosome transfers include induction of centric fusion of unpaired wheat and foreign chromosomes by double monosomy (breakage and reunion) (Friebe et al., 2005; Liu et al., 2011, 2016) or radiation of seeds or pollen to induce random chromosome breaks and reunions (Sears, 1956; Liu et al., 1999). At least 15 cataloged *Pm* genes have been transferred into wheat from wild wheat relatives, including *Pm13* and *Pm66* from *Ae*. *longissima* Schweinf. & Muschl. (2*n* = 2*x* = 14, S^l^S^l^), *Pm29* probably from *Ae*. *geniculata* Roth. (2*n* = 4*x* = 28, U^g^U^g^M^g^M^g^), *Pm57* from *Ae*. *searsii* Feldman & Kislev ex Hammer (2*n* = 2*x* = 14, S^s^S^s^), *Pm7, Pm8, Pm17, Pm20*, and *Pm56* from *Secale cereale* L. (2*n* = 2*x* = 14, RR), *Pm21*(= *Pm31*), *Pm55, Pm62* and *Pm67* from *Dasypyrum villosum* (L.) P. Candargy (2*n* = 2*x* = 14, VV), *Pm51* from *Thinopyrum elongatum* (Host) D. R. Dewey (2*n* = 2*x* = 14, EE), and *Pm2b* from *Agropryron cristatum* (L.) Gaertn. (2*n* = 2*x* = 14, PP). Several of these *Pm* genes, such as *Pm8* on 1RS, and *Pm21* on 6VS, have been widely deployed in wheat resistance breeding programs across the world (He et al., 2018; Li et al., 2020; Zhang et al., 2020).

*Ae*. *longissima* Schweinf. & Muschl. (2*n* = 2*x* = 14, S^l^S^l^), an annual grass species native to the eastern Mediterranean basin is one of the five diploid *Aegilops* species carrying the S, or a modified S genome (Feldman et al., 1995; Friebe et al., 1996). *Ae. longissima* represents a valuable reservoir of genetic diversity for resistance to stem rust, stripe rust, powdery mildew, Septoria glume blotch, and eyespot (Ceoloni et al., 1992; Sheng et al., 2012; Wang et al., 2018; Xia et al., 2018). However, the potential of *Ae*. *longissima* in wheat improvement is far from being fully assessed; only powdery mildew resistance genes *Pm13* (3S^l^S) and *Pm66* (4S^l^S) have been used in wheat breeding programs (Li et al., 2021). Recently, genome sequences of *Ae*. *longissima* accession of TL05 (Li et al., 2022) and AEG-6782-2 (Avni et al., 2022) have been published. These genomic resources will accelerate the exploration and utilization of beneficial genes from this species.

Previously, we identified a Chinese Spring (CS)-*Ae. longissima* 6S^l^#3 disomic addition line TA7548 with resistance to wheat powdery mildew (Xia et al., 2018). In this study, we described the development of 6S^l^#3 recombinants conferring resistance to powdery mildew and the physical mapping of *Pm6Sl* in *Ae. longissima* chromosome 6S^l^#3.

## Materials and Methods

### Plant Material

The plant material used in this study included common wheat CS, CS *ph1b* mutant TA3809 lacking pairing homologous gene *Ph1*, thereby permitting homoeologous recombination, CS-*Ae*. *longissima* 6S^l^#3 disomic addition line TA7548, where a pair of chromosomes 6S^l^#3 from *Ae*. *longissima* was added to CS background, 6S^l^#3 donor *Ae*. *longissima* accession TA1910, CS nulli-tetrasomic stocks N6AT6B (TA3152), N6BT6D (TA3155), and N6DT6A (TA3156), in which the chromosomes 6A, 6B and 6D pairs were respectively replaced by homoeologous pairs 6B, 6D, and 6A. The number following the chromosome designation (6S^l^#3) indicated the origin of the alien chromosome derived from different *Ae. longissima* accessions (Raupp et al., 1995). All materials were provided by the Wheat Genetics Resource Center (WGRC), Kansas State University, USA, and increased in China at the Experimental Station of Henan Agricultural University.

### Development of Populations Segregating for Chromosome 6S^l^#3

Two populations segregating for 6S^l^#3 were developed. Population 1 produced by crossing CS monosomic 6A (CSM6A, 20W″ + 6A′) with TA7548 (21W″ + 6S^l^#3″) was used to develop compensating Robertsonian translocations (RobTs) involving wheat chromosome 6A and 6S^l^#3. F_1_ plants with 42 chromosomes and positive for 6S^l^#3-specific markers were selected and self-pollinated.

Population 2 used to develop 6S^l^#3 recombinants was derived from the hybrid of CS *ph1b* mutant TA3809 crossed with TA7548. F_2_ individuals with homozygous *ph1b* and monosomic 6S^l^#3 were selected using the *ph1b-*specific marker ABC302.3 (Wang et al., 2002) and 6S^l^#3-specific markers. Self-pollinated progenies of these plants were used to screen putative 6S^l^#3 recombinants.

Individual plants of both populations were screened by a few 6S^l^#3-specific molecular markers to select the plants showing marker-disassociation as putative recombinants involving 6S^l^#3 and then verified by cytogenetic and molecular marker analyses.

### Preparation, Sequencing, and Alignment of an *Ae*. *longissima* Full-Length cDNA Library

Preparation, sequencing and alignment of an *Ae*. *longissima* full-length cDNA library was performed by BGI Genomics (Shenzhen, China) with the Pacific Bioscience Sequel platform (Pacific Biosciences, Silicon Valley, California, USA). Briefly, the second leaves from *Ae*. *longissima* TA1910 were collected at 0, 12, 24, and 48 h post-inoculation with *Bgt* isolate E26 and equally mixed for total RNA extraction using a mirVana miRNA Isolation Kit (Cat. No. AM1561, Ambion, Thermo Fisher Scientific, Waltham, MA, USA) following the manufacturer's protocol. Total RNA was converted to first-strand cDNA using a SMARTer PCR cDNA Synthesis Kit (Cat. No. 634925, Clontech, Takara Biomedical Technology (Beijing) Co., Ltd., Beijing). After PCR optimization, a large-scale PCR was performed to synthesize second-strand cDNA. After another large-scale PCR, the DNA was ready for library template preparation by PacBio SMRTbell; selected fragments > 4 kb were sequenced by Pacific Biosciences Sequel. Sequencing data were processed by Single-Molecule, Real-Time (SMRT) sequencing analysis through reads of insert, classify, and cluster to obtain consensus full-length isoforms. Isoforms that could not be aligned to any database were predicted by TransDecoder version v3.0.1 (https://transdecoder.github.io).

### Molecular Marker Analysis

Fifty-three markers were used in this study, of which 24 were 6S^l^#3-specific markers developed from full-length cDNA sequences of TA1910, and the other 29 simple sequence repeat (SSR) markers specific for wheat chromosomes 6A (15), 6B (8), and 6D (6) were obtained from GrainGenes. SSR marker details are listed in Supplementary Table S1. The genomic positions of the markers of 6S^l^#3-specificity were ordered using blastn alignment against both the CS reference genomic sequence (Wheat_IWGSC_RefSeq_v2.1) (Zhu et al., 2021) and *Ae*. *longissima* accession TL05 reference sequences (Li et al., 2022).

Genomic DNA (gDNA) was isolated from 5 to 10 cm segments of young leaves with a DNeasy Plant Mini Kit (Qiagen, Cat No. 69104) following the instructions. PCR reactions were conducted in a 15-μl volume containing 2.0 μl template gDNA (100 ng/μl), 1.0 μl of each primer (5.0 μmol/l), 7.5 μl Taq MasterMix (CW Bio Inc., China) and 3.5 μl ddH_2_O. Using 6S^l^#3-specific primers, PCR amplifications were performed by Touchdown63 and by F50SSR using SSR markers (Liu et al., 2017). PCR products of 6S^l^#3-specific markers and SSR markers were resolved in 1.5% and 3.0% agarose gels and visualized by ethidium bromide staining under UV light.

### Cytogenetic Analysis

Collection and nitrous oxide treatment of root tips, squash preparations, and genomic *in situ* hybridization (GISH) were performed following protocols described by Liu et al. (2017). *Ae*. *longissima* gDNA was labeled with fluorescein-12-dUTP. Common wheat CS gDNA was used as blocking. The ratio of gDNA of *Ae*. *longissima* to CS was 1:120. Fluorescence *in situ* hybridization (FISH) was performed with eight oligonucleotide probes, of which six were 6-carboxytetramethylrhodamine (TAMRA)-modified oligonucleotides (pAs1-1, pAs1-3, pAs1-4, pAs1-6, AFA-3, and AFA-4) and displayed red signals, and the other two were 6-carboxyfluorescein (FAM)-modified oligonucleotides (pSc119.2-1 and (GAA)_10_), which fluoresced green (Du et al., 2017; Huang et al., 2018). After hybridization and slide washing, a drop (25–30 μl) of Vectashield mounting medium containing 1 μg/ml DAPI (Vector Laboratories Inc, Burlingame, CA, USA) was added to each slide and then covered with a 24 × 30 cm glass coverslip. Fluorescent images were observed under a Zeiss Axio Scope A1 fluorescence microscope (Jena, Germany) and captured with an AxioCam MRc5 CCD camera. Images were further processed with Adobe Photoshop CS3 (Version 10.0.1) (Adobe Systems Inc., San Jose, CA, USA).

### Powdery Mildew Evaluation

Thirty single-pustule-derived *Bgt* isolates were collected from different regions in China and used to test TA7548. Isolate E26, kindly provided by the Nanjing Agricultural University and locally maintained, was used to test the 6S^l^#3 derivatives. The remaining 29 isolates were maintained at the University of Yantai, Shandong Province. The preparation of plant materials and *Bgt* inoculation followed protocols was described by Li et al. (2020). Infection types were recorded on a 0–4 scale at 10 days post inoculation, while conidia were fully developed on the first leaves of susceptible control CS. Plants with ITs of 0–2 were considered to be resistant, whereas those with ITs of 3–4 were susceptible (Shi et al., 1987).

## Results

### Response of TA7548 to a Panel of *Bgt* Isolates

Thirty *Bgt* isolates were used to test the *Bgt* response of TA7548 together with CS as a susceptible control when the first leaves were fully expanded. Seedling reactions 10 days post-inoculation indicated that 28 of 30 *Bgt* isolates were avirulent on TA7548, with ITs ranging from 0 to 2, and the other two isolates were virulent on TA7548 with ITs 3 and 4. CS, the susceptible control, was highly susceptible to all *Bgt*-isolates, with ITs 3-4 (Table 1). Thus, the gene(s) on chromosome 6S^l^#3, designated as *Pm6Sl*, confers resistance to multiple *Bgt*-isolates.

**Table 1:** Responses of TA7548 to 30 *Bgt* isolates.

***Bgt*-isolates**	**IT**		***Bgt*-isolates**	**IT**	
	**CS**	**TA7548**		**CS**	**TA7548**
A10	4	0	Y04	4	3
E05	4	0	Y15	4	1
E09	3	0	Y06	4	0
E31	3	0	Y07	4	0
E18	3	0	Y08	4	0
A3	4	0	Y09	4	0
E23-1	3	0	Y10	4	2
E20	3	0	Y11	4	2
E21	4	0	Y14	4	0
E26	4	0	Y16	4	0;
E32	4	0	Y17	4	0
E23	4	0	Y18	3	0
Y01	4	0	Y21	4	0
Y02	4	4	B18	4	0
Y03	3	0	GY-KDZ-1	4	1

### *Ae*. *longissima* Full-Length cDNA Sequences

Sequenced by the Pacific Bioscience Sequel platform generated 2,136,574,821 bp CCS (reads of insert) data (1,161,885 reads), of which, 685,686 (59.01%) reads were full-length non-chimeric, with a mean length of 1,494.7 bp. After clustering and polishing the consensus with Quiver and removing redundancies, a total of 69,907 high-quality consensus isoforms were identified, covering a total length of 113,954,143 bp, with a mean length of 1,630 bp and N50 of 1,920 bp. The isoforms were further analyzed using TransDecoder software to identify candidate coding regions. The longest open reading frame (ORF) was then selected and blasted to the SwissProt and Hmmscan databases to search for Pfam protein homology sequences for CDS prediction, leading to the prediction of 62,761 (71,013,186 bp) CDS with lengths ranging from 297 to 5,913 bp were predicted.

### Development of 6S^l^#3-Specific Markers

By blasting the full-length sequences of 62,761 predicted CDS of *Ae*. *longissima* to CS reference genomic sequences (IWGSC RefSeq v2.1, IWGSC, 2021), a total of 8,461 (13.48%) CDS were uniquely assigned to homoeologous group 6. One hundred and thirty-four unique CDS with similarities of 80–90% to wheat group 6 genomic sequences were selected for further PCR primer design. Based on a comparison of these CDS sequences with those of CS, 134 PCR primer pairs were designed. The comparison of amplification of CS and CS-*Ae. longissima* 6S^l^#3 disomic addition line TA7548 led to the identification of 24 (17.91%) markers specific to chromosome 6S^l^#3 (Table 2).

**Table 2:** *Ae. longissima* 6S^l^#3 specific markers developed in this study.

**Maker name**	**Position (Mb)**	**Forward primer (5'-3')**	**Reverse primer (5'-3')**	**Tm (**°**C)**	**Product size (bp)**
Ael69501	0.37	TGGGCCTTTGAGTAGAGC	ACTGACCACGATAGGTTTTT	63	197
Ael69148	3.97	TCGCCACCGCAGACCTT	TGGCTGGCTGCTTTGGG	63	111
Ael60640	7.21	GACCTGAAAGCGTCATACC	TTACAATACCGACGACAATG	63	410
Ael68035	9.04	GATTGCATGAATTATACTCTGG	AACAGACCCTCAACCGAAA	63	122
Ael67319	202.09	TATGTCTAGTGTATCTGTACGTGGC	ACTCATGTACAAGAGTAATCAGCAC	56.5	172
Ael63185	383.52	GAGCAGGCGTAGCCTCAGGAGCCTC	TTTTCTCTACCTAAGTGCATGGAAG	56.5	212
Ael65131	423.79	ACAGAACAATCGATGCGTAC	TCCTGTTACAAGATGCCCAG	63	543
Ael56039	433.79	CGAGAAAGGTGTATTGCCG	TCCAAACTGAATCCCAACA	63	305
Ael59504	440.43	AGTGGAACGCAGTGGCAA	CGGCGGGAGTAGTAGATTT	63	212
Ael68886	442.72	TGGCAGTGGCGGAGGAC	TTCTGCCATGACCTACTGACC	63	268
Ael63597	504.91	GCCGCTCTGTTTTACCTTC	AGATTTAGCAGCACTGTTCG	63	118
Ael59927	506.99	TTTGGATCGCGTGGGCA	GTGCACCGGCCAAGTAATG	63	195
Ael58062	511.11	ATCGTCATTCGTCGCCCT	GTCCACGCGCATCAGCA	63	376
Ael63981	526.76	GTCCGTCTACCTACCCTAGC	CAAAGAGGTACGGTACAAGC	63	168
Ael68067	527.64	ATCGTGGGAGAGGTGCAG	TTGGGTCGTGATCCATTG	63	408
Ael58468	580.44	CTGATCGACCGACCGATT	ATGCACATGGGCAAGAAC	63	249
Ael60368	580.44	CACAGAGAGGGAGAGAGATACT	AGGCCACCTGATACCAAC	63	143
Ael59300	580.44	GCTTGTCGACCGGGAGG	CTCTCATGCGTCTGCGTATT	63	240
Ael55917	608.24	CTCTCCGTCGTCGTCATG	TTCACCGGTTATTGACATCA	63	390
Ael58410	616.84	CATCGGGGGAGCGGTAC	CCCTAGCTACCAGATCACCC	60	159
Ael58120	620.73	TGTTCTGAGCATCACCATAC	TCATGCGAGTACATCAACC	55	261
Ael64341	621.98	CAAGTCTGTCAGCGTCTCG	CACAAGAGAGGAGGAATACGT	55	463
Ael57699	659.64	TACTGCAATGAGCTCAAGTCCAACA	TCGAGGAACACAACATCTTGCTGTT	67	183
Ael42958	659.64	GCCAACTGAGGTTAGTTTCGTTAAT	CTCACCTCACTAATCAGCCTTCACC	65	536

*Position determined by synteny comparison with Ae. longissima TL05 6S^l^ reference genomic sequences. Tm: annealing temperature*.

### Identification of RobTs Involving 6S^l^#3

**Figure 1:**
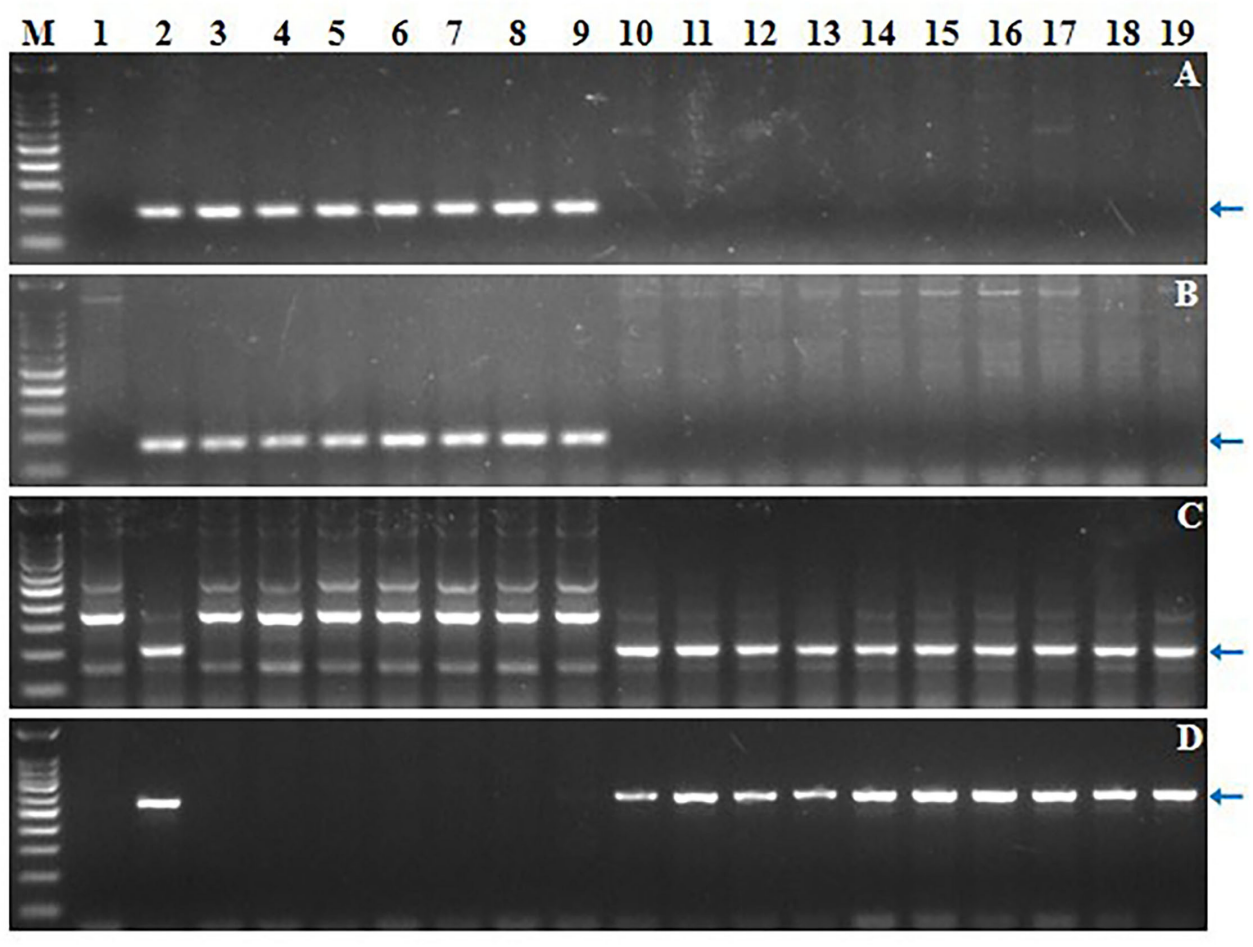
Screening of 17 CS-*Ae. longissima* 6S^l^#3 RobTs by molecular marker analysis. **(A–D)** Electrophoresis patterns of 6S^l^#3-specific markers Ael69501, Ael67319, Ael63185 and Ael42958, respectively. M: 100 bp DNA Ladder Marker; 1: common wheat CS; 2: CS-*Ae. longissima* 6S^l^#3 disomic addition line TA7548; 3-19: the newly developed CS-*Ae. longissima* 6S^l^#3 RobTs and telosomes. Arrows pointed to the polymorphic bands of the chromosome 6S^l^#3-specific molecular markers.

**Figure 2:**
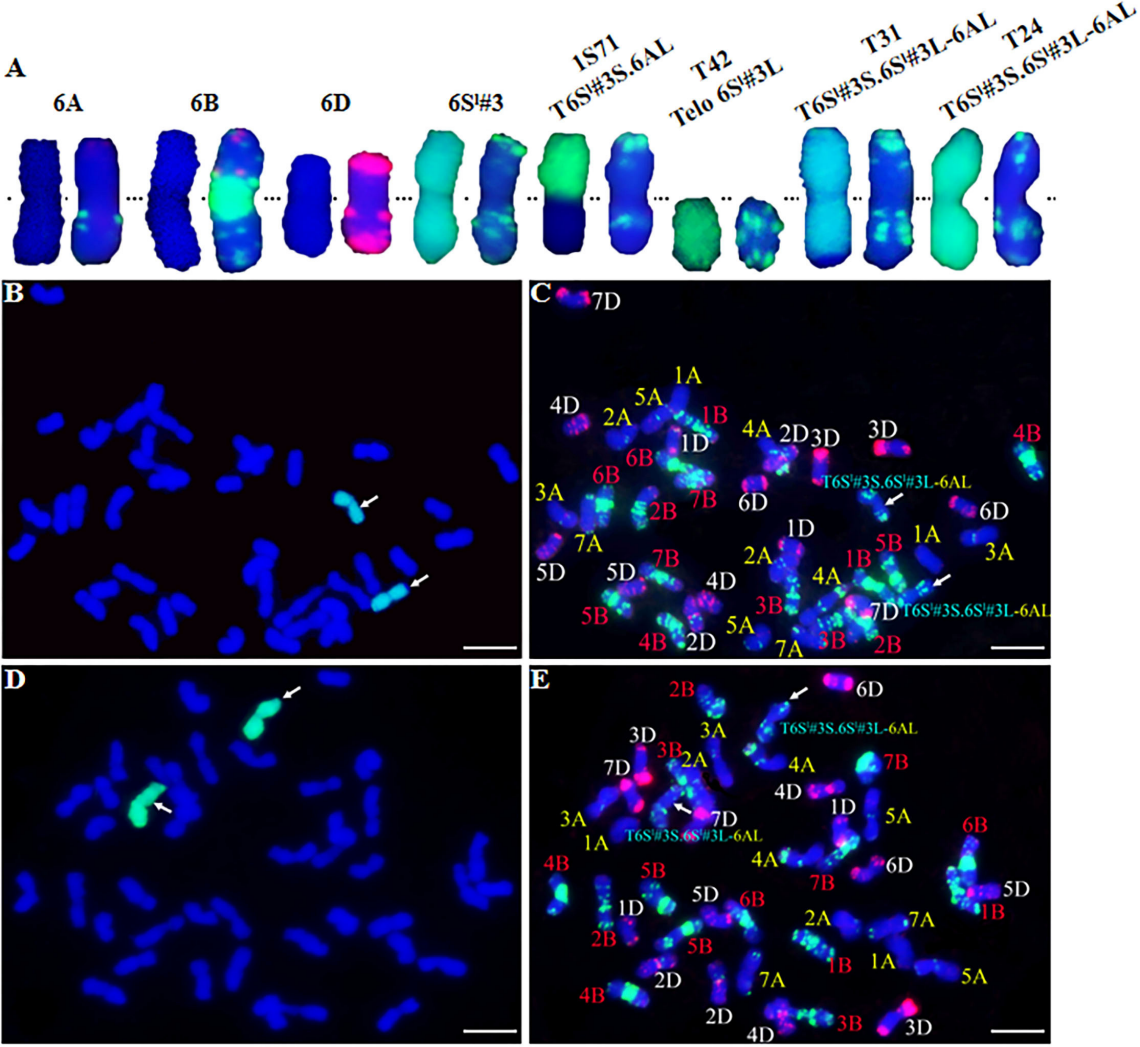
GISH and FISH analysis of CS-*Ae. longissima* 6S^l^#3 introgression lines. **(A)** From left to right, chromosome pairs of 6A, 6B, 6D, 6S^l^#3, T6S^l^#3S.6AL (1S71), 6S^l^#3L telosome (T42), T6S^l^#3S.6S^l^#3L-6AL (T31), and T6S^l^#3S.6S^l^#3L-6AL (T24). The left and right chromosome of each pair is GISH and FISH, respectively. **(B–E)** GISH and FISH patterns of T31 **(B,C)** and T24 **(D,E)**. Arrows indicated the recombined chromosomes involving 6S^l^#3. *Ae. longissima* chromatin is visualized by green florescence and wheat chromosomes are counterstained with DAPI and fluoresced blue in GISH. In FISH, TAMRA-modified oligonucleoties (pAs1-1, pAs1-3, pAs1-4, pAs1-6, AFA-3 and AFA-4) are in red color, FAM-modified pSc119.2-1 and (GAA)_10_ are in green color. Scale bar = 10 μm.

**Figure 3:**
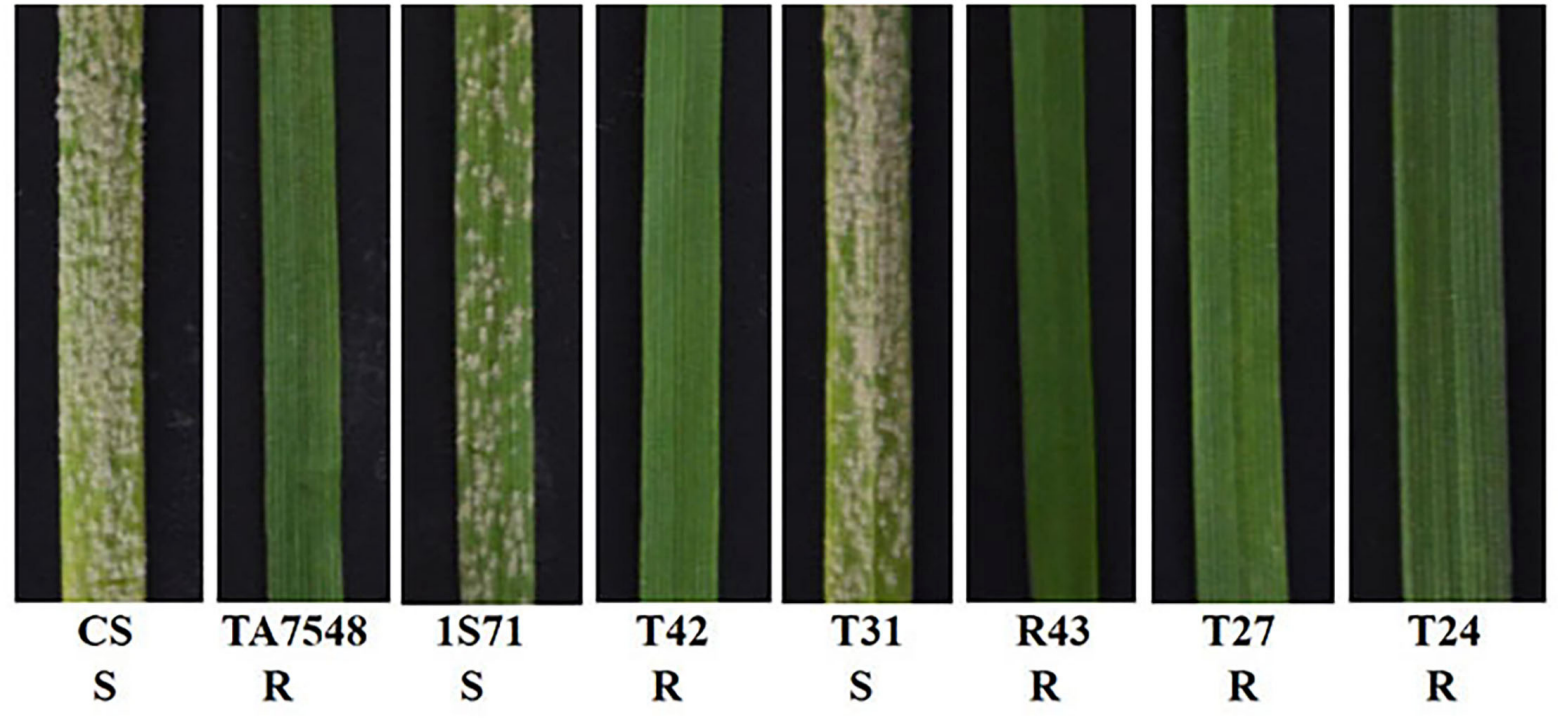
Powdery mildew resistance evaluation of 6S^l^#3 recombinants. 1S71: CS-*Ae. longissima* RobT T6S^l^#3S.6AL; T42: 6S^l^#3L telosome line; T31: CS-*Ae. longissima* T6S^l^#3S.6S^l^#3L-6AL recombinant; R43: CS-*Ae. longissima* T6BS.6BL-6S^l^#3L recombinant; T27: CS-*Ae. longissima* Ti6AS.6AL-6S^l^#3L-6AL recombinant; T24: CS-*Ae. longissima* T6S^l^#3S.6S^l^#3L-6AL recombinant. R: resistant to powdery mildew, S: susceptible to powdery mildew.

Four markers mapped to each arm of 6S^l^#3 were used to select putative RobTs involving 6S^l^#3 in Population 1. Markers Ael69501 and Ael42958 were distally located at opposite ends of 6S^l^#3, whereas Ael67319 and Ael63185 occupied intermediate positions in the short and long arms. Plants lacking either the short arm- or long arm-specific marker(s) were selected as putative 6S^l^#3 RobTs. From 608 plants in population 1, 17 (2.80%) showing disassociations of markers were selected as putative RobTs or having telosomes (Telos) for either 6S^l^#3S or 6S^l^#3L. Seven plants lacked long arm markers and 10 lacked short arm markers (Figure 1). GISH and FISH analyses of the seven plants lacking the long arm markers identified a compensating RobT T6S^l^#3S.6AL (1S71), whereas the remaining plants were monotelosomic 6S^l^#3S (Figure 2). The 10 plants with only long arm markers were all monotelosomic 6S^l^#3L (represented by T42) (Figure 2). A powdery mildew test using *Bgt*-isolate E26 showed that the RobT T6S^l^#3S.6AL and the six monotelosomic 6S^l^#3S were susceptible, whereas all 10 monotelosomic 6S^l^#3L were resistant with IT of 0 (Figure 3). Thus, *Pm6Sl* was located in the long arm of chromosome 6S^l^#3.

### Development of CS-*Ae*. *longissima* 6S^l^#3L Recombinants

**Figure 4:**
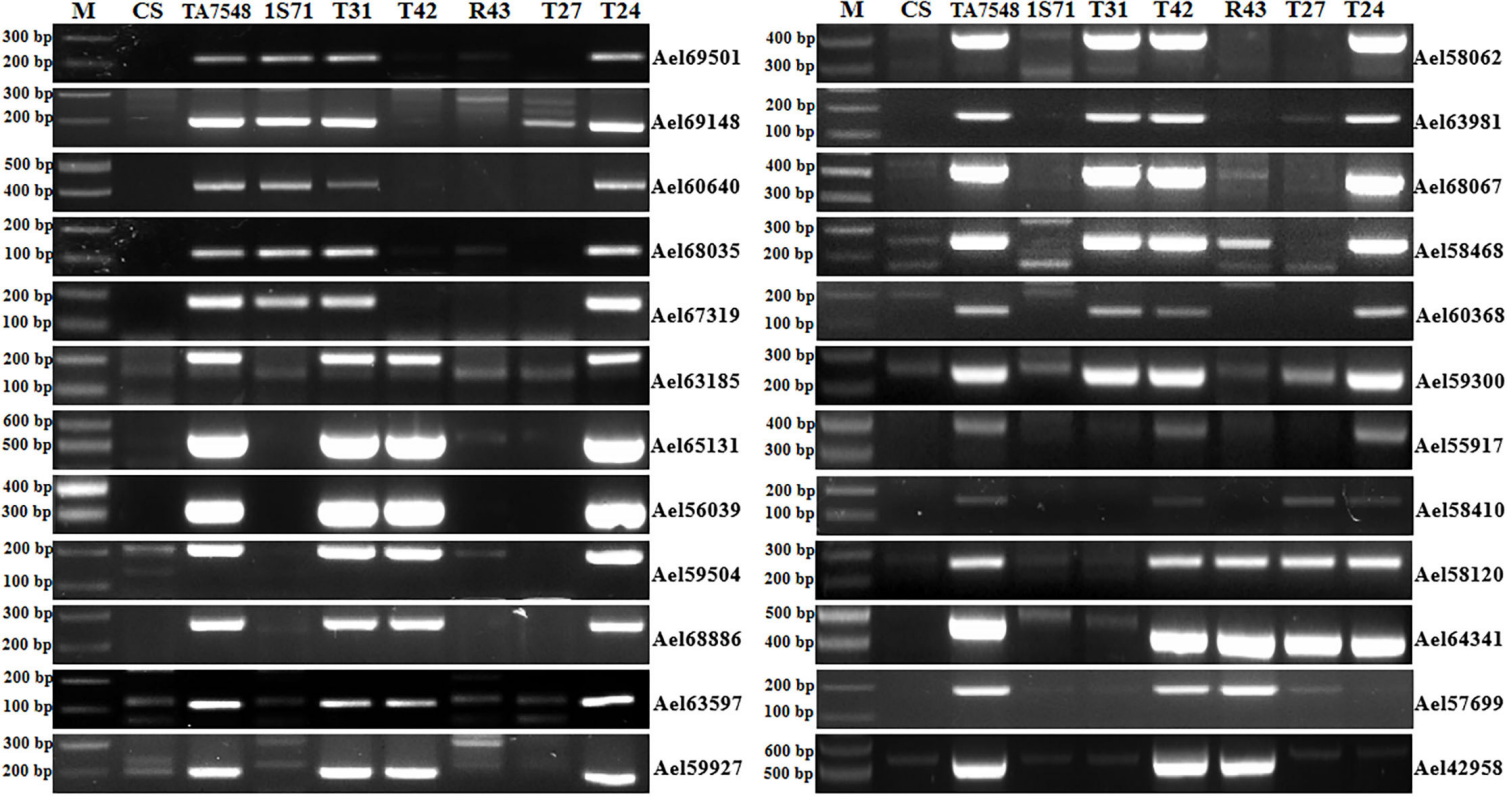
PCR patterns of 6S^l^#3 recombinants using 6S^l^#3-specific markers. M: 100 bp DNA ladder marker.

In order to develop CS-*Ae*. *longissima* recombinants with breakpoints at 6S^l^#3L where *Pm6Sl* resides, four markers specific for 6S^l^#3L were used to select putative recombinants in Population 2. Two markers (Ael65131 and Ael56039) were near the centromere of 6S^l^#3L, whereas the other two, Ael58410 and Ael42958, were located at the distal region of 6S^l^#3L. Fourteen plants (1.76%) displayed disassociations with the four markers and were selected as putative 6S^l^#3L recombinants. These putative 6S^l^#3L recombinants were further characterized using 24 6S^l^#3-specific markers. Based on the presence of different markers, the 14 plants were grouped into four types: T31, T24, R43, and T27 (Figure 4).

T31 type lost six proximal and distal markers of 6S^l^#3L (Ael55917, Ael58410, Ael58120, Ael64341, Ael57699, and Ael42958), which covered a distal segment of <81.77 Mb. T24 plants had all 6S^l^#3-specific markers except two-terminal markers (Ael57699 and Ael42958), both at 659.64 Mb. The R43 type presented only four distal markers (Ael58120, Ael64341, Ael57699, and Ael42958) of 6S^l^#3L covering a segment of <45.37 Mb. T27 was positive for only three distal markers of 6S^l^#3L covering a segment of <51.40 Mb; however, both terminal markers of 6S^l^#3L (Ael57699 and Ael42958) at 659.64 Mb were absent (Figure 4). Chromosome 6S^l^ segments in both recombinants R43 and T27 were < 8% of 6S^l^ genomic length.

### Cytogenetic Analysis of 6S^l^#3L Recombinants

GISH and FISH were performed to identify chromosome recombinants involving 6S^l^#3L. GISH showed that type T31 contained a recombined 6S^l^#3 chromosome with a terminal wheat segment consistent with 6AL (Figures 2B,C). Therefore, the T31 recombinants were identified as T6S^l^#3S^.^6S^l^#3L-6AL. Neither GISH nor FISH identified a wheat segment replacing distal 6S^l^#3L in the T24 type despite positive indications from marker analysis (Figures 2D,E). The results indicated that those plants had a small distal segment of wheat replacing the 6S^l^#3L counterpart. GISH of both R43 and T27 types failed to detect the green color for *Ae*. *longissima* chromatin, and FISH also failed to detect a difference from CS. This confirmed that any 6S^l^#3 segments in these lines should be small and beyond the resolving power of these methods.

### Characterization of 6S^l^#3L Recombinants by SSR Markers

The combined GISH, FISH patterns, and 6S^l^#3-specific marker analyses indicated that all putative recombinants contained 6S^l^#3L segments of various sizes, whereas the identities of the wheat segments in the other groups except T31 were unresolved. SSR markers were used to further characterize those 6S^l^#3L recombinants. Twenty-nine SSR markers specific to chromosomes 6A (15), 6B (8) and 6D (6) were selected to perform PCR amplification (Supplementary Table S1). Compared with CS and TA7548, different PCR products were amplified from T31, T27, and T24 for 6A-SSRs, and R43 for 6B-SSRs.

**Figure 5:**
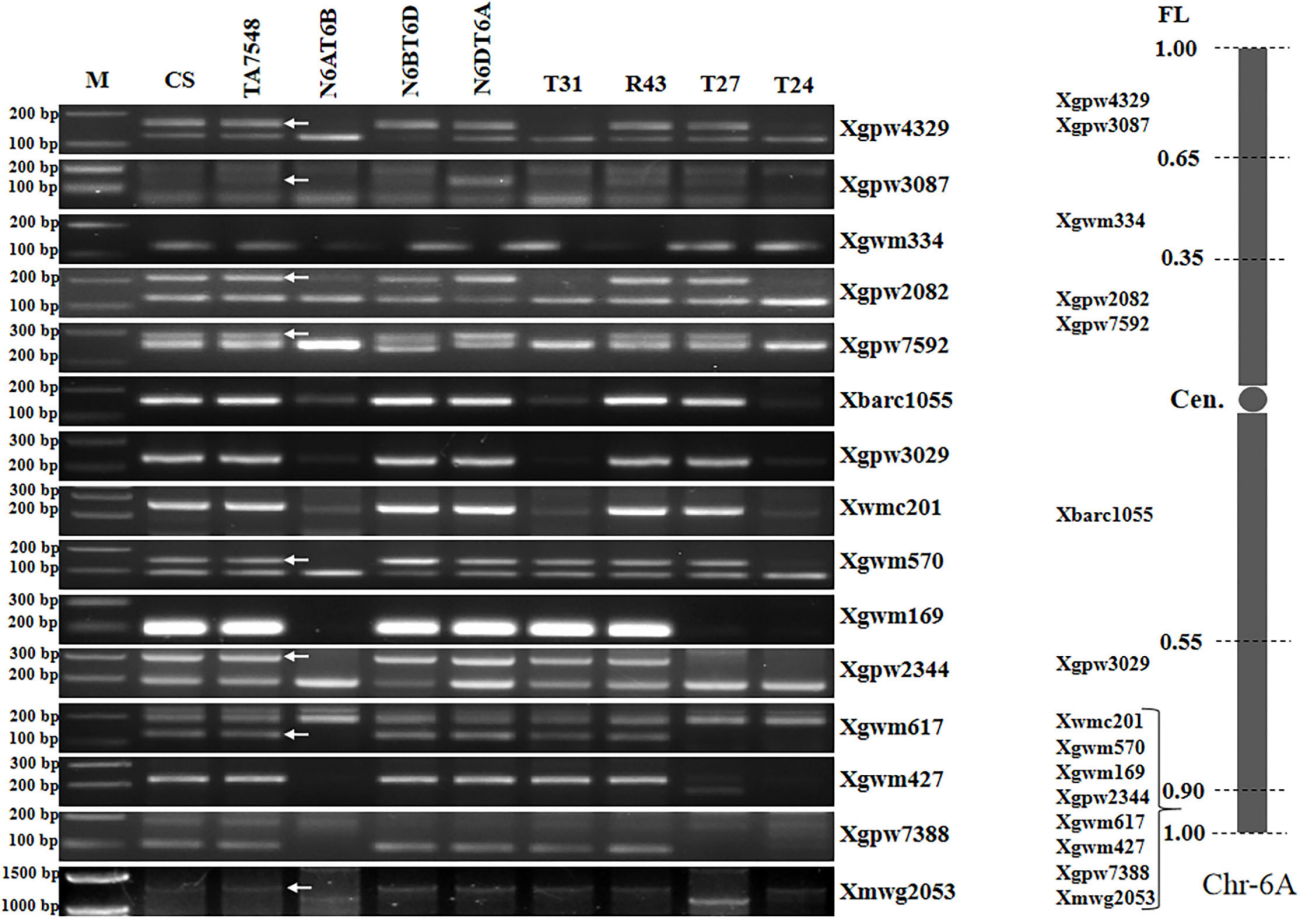
Molecular markers analysis of 6S^l^#3 recombinants using wheat 6A SSR markers. Arrows pointed to 6A-specific bands when two or more bands were amplified using a single primer pair. M: 100 bp DNA ladder marker.

Analyses of 15 6A-specific SSR markers displayed that recombinants of type T31 lacked all 6A-specific SSR markers except seven distal markers in chromosome bin 6AL8-0.90-1.00 (Figure 5; Supplementary Table S2), indicating that T31 lines had recombined chromosomes containing only small segments of 6AL, thus designated as T6S^l^#3S.6S^l^#3L-6AL (Supplementary Figure S1). Type T24 presented SSR patterns similar to those of T31 and was also designated as T6S^l^#3S.6S^l^#3L-6AL, but the wheat segment was smaller with just distal SSR marker Xmwg2053 (Figure 5; Supplementary Table S2). T27 recombinants lacked six distal markers in chromosome bin 6AL8-0.90-1.00, whereas they retained two proximal markers (Xwmc201 and Xgwm570) (Figure 5; Supplementary Table S2). By combining 6S^l^#3 specific marker and SSR markers analyses, T27 should be an interstitial recombinant formed by proximal 6S^l^#3L segments replaced 6AL counterparts, thus designated as Ti6AS.6AL-6S^l^#3L-6AL (Supplementary Figure S1).

**Figure 6:**
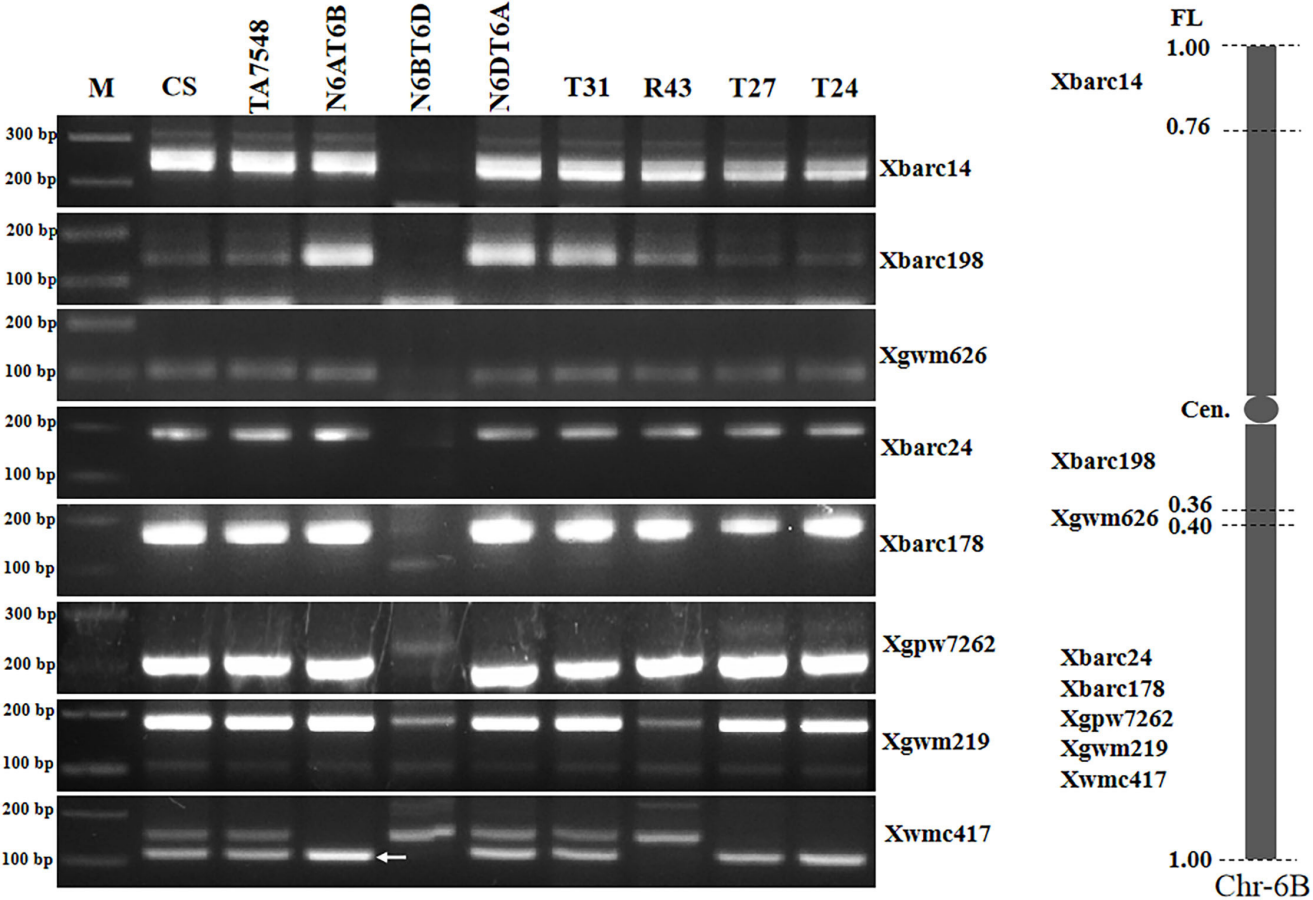
Molecular markers analysis of 6S^l^#3 recombinants using wheat 6B SSR markers. Arrows pointed to 6B-specific bands when two or more bands were amplified using a single primer pair. M: 100 bp DNA ladder marker.

PCR patterns of eight 6B-specific SSR markers displayed that type R43 recombinants were negative for two of five markers (Xgwm219 and Xwmc417) in chromosome bin 6BL5-0.40-1.00 but positive for the remaining six markers (Figure 6; Supplementary Table S2). We concluded that the recombined chromosomes in R43 were formed by the 6S^l^#3L distal segments substituting for distal counterparts of 6BL. Thus, R43 was designated T6BS.6BL-6S^l^#3L (Supplementary Figure S1).

### Physical Mapping of *Pm6Sl*

**Figure 7:**
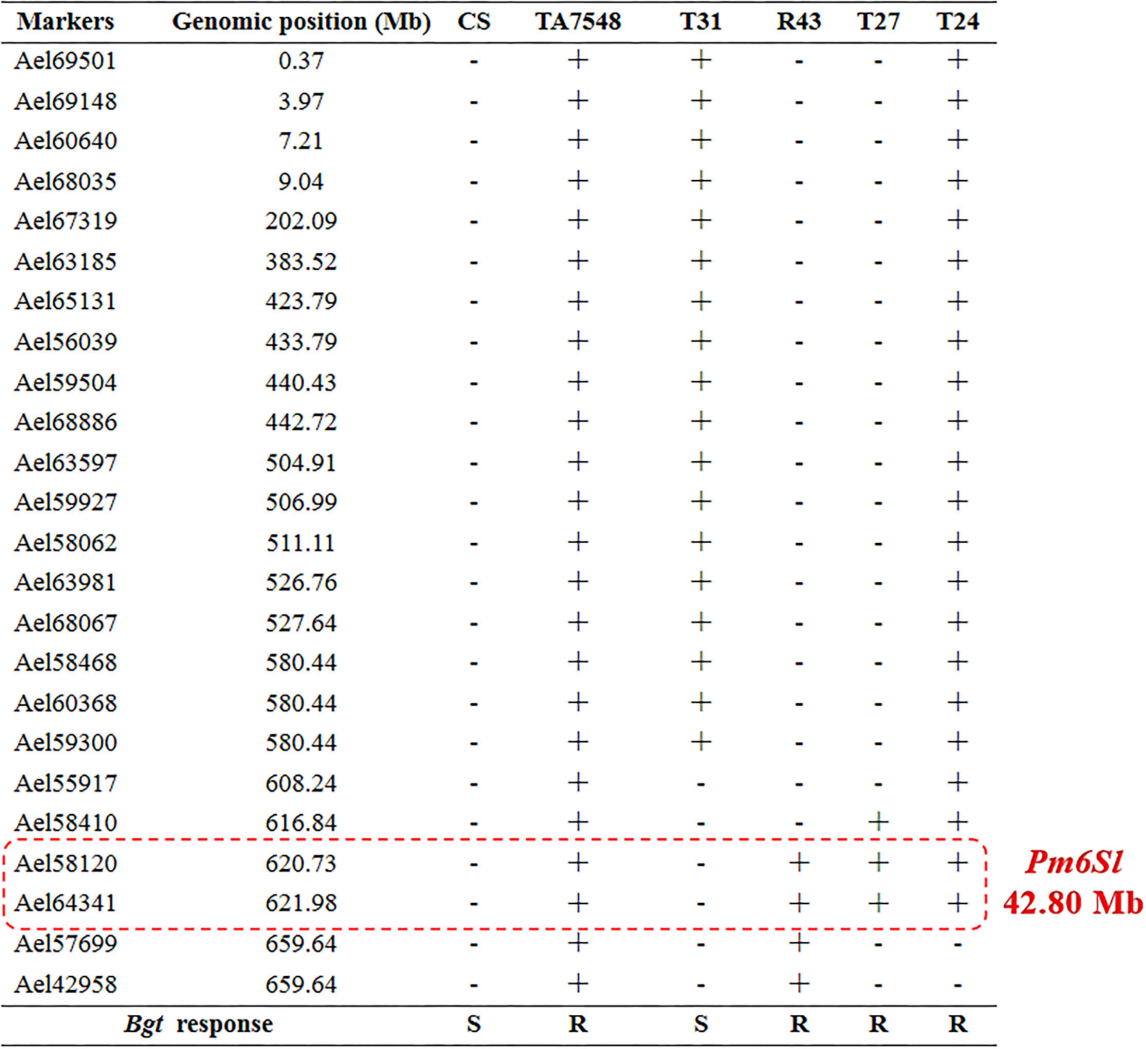
Physical mapping of gene *Pm6Sl*. “+” indicated the presence of 6S^l^#3-specific molecular markers, “**–**” indicated the absence of 6S^l^#3-specific molecular marker. R indicated resistance to powdery mildew, S indicated susceptible to powdery mildew.

The 6S^l^#3L recombinants, together with susceptible control CS and resistant control TA7548, were assayed by inoculation of the *Bgt*-isolate E26. Infection types at 10 days post-inoculation indicated that recombinants of T24, R43, T27, and TA7548 were resistant (IT 0), whereas T31 plants and CS were susceptible (IT 4) (Figure 3). By integration of the *Bgt*-responsive assay and molecular marker analyses, *Pm6Sl* was physically mapped to a distal interval between markers Ael58410 and Ael42958 in the long arm of 6S^l^#3, which spanned 42.8 Mb in *Ae*. *longissima* TL05 reference sequence (Figure 7).

### Identification of Specific Markers for *Pm6Sl* in Wheat Genetic Backgrounds

**Figure 8:**
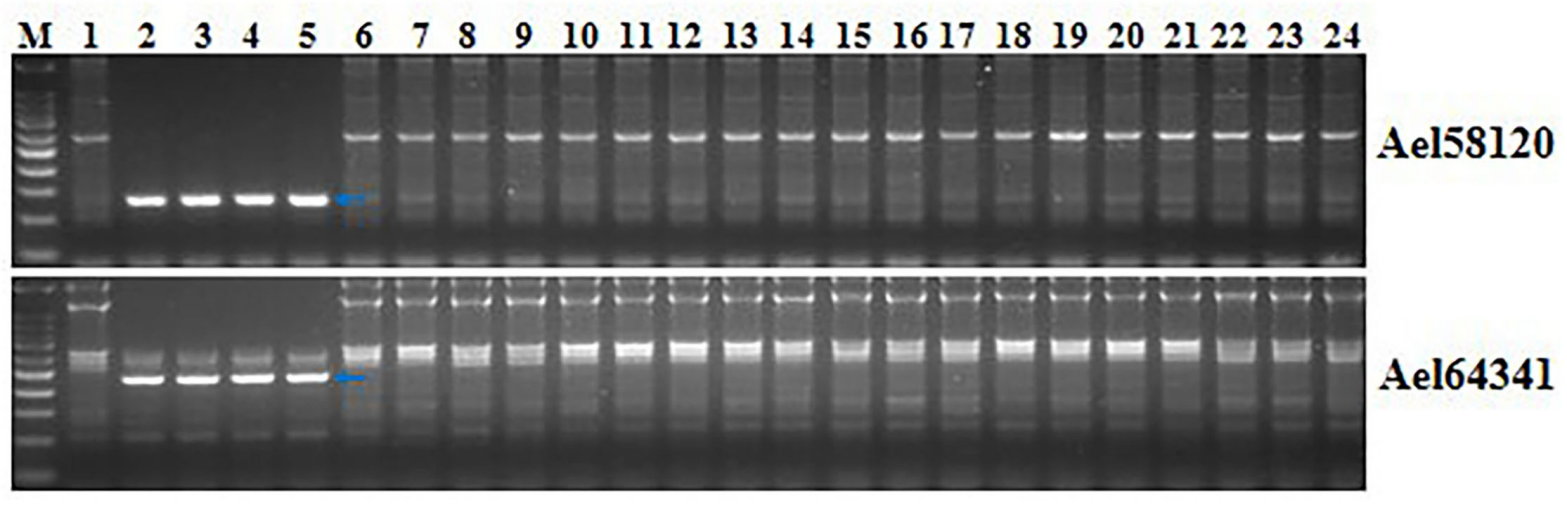
Validation of the usefulness of two markers Ael58120 and Ael64341 closely linked to *Pm6Sl*. M: 100 bp DNA ladder; 1: CS; 2: CS-*Ae*. *longissima* 6S^l^#3 disomic addition line TA7548; 3-5: 6S^l^#3 recombinants R43, T24 and T27 with resistance to powdery mildew; 6: Bainong 207; 7: Bainong 64; 8: Aikang 58; 9: Zhoumai 16; 10: Zhoumai 18; 11: Zhoumai 22; 12: Zhoumai 28; 13: Pingan 0518; 14: Pingan 602; 15: Pingan 901; 16: Jimai 22; 17: Yanzhan 4110; 18: Shengxuan 6; 19: Yangmai 5; 20: Nannong 9918; 21: Linxuan 101; 22: Hua 5; 23: Ningmai 3; 24: Xinong 979. Arrows pointed to the polymorphic bands of the respective molecular markers linked to *Pm6Sl*.

Two markers developed in this study, Ael58120 and Ael64341, were close to *Pm6Sl*. PCR amplification was performed to determine whether they were diagnostic with four *Pm6Sl* stocks, including CS-*Ae*. *longissima* 6S^l^#3 disomic addition line TA7548, recombinant R43, T24, and T27, and 23 wheat lines. The target bands were amplified from *Pm6Sl* stocks but were absent in those wheat varieties not containing *Pm6Sl* (Figure 8). Thus, both PCR markers can be used to assist the selection of *Pm6Sl* plants in the hybrid progeny of these *Pm6Sl* stocks in breeding programs.

## Discussion

*in situ* hybridization (GISH and FISH) provides a highly efficient approach to resolving alien chromosome segments in wheat (Li et al., 2019). The combined application of both techniques can display the size of both donor and recipient wheat chromosomes and the number and position of breakage points. However, the resolution of GISH is about 25 Mb (Mukai et al., 1993). Similarly, FISH can also fail to resolve chromosomes or segments unless there are presented abundant signal bands. In contrast, transcriptome sequencing can quickly and economically yield numerous sequences to facilitate the rapid and efficient development of molecular markers covering all the genomes of wild wheat relatives. Combined GISH/FISH with chromosome-specific markers has been widely used to accelerate the detection of alien chromatin harboring useful genes in wheat introgression and breeding programs (Wang et al., 2018; Zhang et al., 2019; Li et al., 2020). *Ae*. *longissima* belongs to the section *Sitopsis* in the genus *Aegilops*. The S or modified S genomes of the section are genetically related to the wheat B and D genomes, thus resolving small segments of *Ae*. *longissima* chromatins introgressed into wheat by GISH is challenging. In this study, we successfully identified RobT lines with centric fusion T6S^l^#3S.6AL (1S71), telosomes 6S^l^#3S and 6S^l^#3L, and recombinant T6S^l^#3S.6S^l^#3L-6AL (T31) by combining GISH and FISH, whereas other recombinants were unresolved. By integrating both 6S^l^#3-specific and wheat SSR marker analyses, we identified recombinants T27 (Ti6AS.6AL-6S^l^#3L-6AL), T24 (T6S^l^#3S.6S^l^#3L-6AL), and R43 (T6BS.6BL-6S^l^#3L).

Mapping and cloning of genes from wild species still face tough challenges due to *Ph* gene-suppressed recombination between alien chromosomes and their wheat homoeologous counterparts, lack of reference genomic sequences for those wild species, and insufficient markers specific to alien chromosomes. In this study, we constructed a full-length cDNA sequence database of *Ae*. *longissima* accession TA1910, and designed PCR primers based on isoform sequence comparisons with CS reference genomic sequences. About 18% of molecular markers designed in the study gave polymorphic bands to distinguish *Ae*. *longissima* 6S^l^#3 chromatin from wheat chromatin. This was much more efficient than the EST-PCR, SSR, and mapped-flcDNA-based marker development that we reported previously (Liu et al., 2016). By marker analysis and tests of powdery mildew response, *Pm6Sl* was located at the distal interval of 42.80 Mb flanked by markers Ael58410 and Ael57699 in the long arm of 6S^l^#3. The resistant recombinants with small 6S^l^#3 segments and the full-length cDNA sequence database of TA1910 developed in this study will help future fine mapping and cloning of *Pm6Sl*.

The transfer of favorable genes from wild relatives of common wheat is an effective approach to broadening the genetic base of modern wheat. Homoeologous recombination-based transfer is currently the best way to introgress desirable genes from wild species to common wheat. However, homoeologous recombination between alien chromatin and wheat homoeologous counterparts was restrained in the presence of *Ph* genes (Gyawali et al., 2019). The deletion mutant (*ph1b*) of the pairing homologous gene *Ph1* at 5BL is mostly deployed to promote homoeologous recombination between wild species and wheat. A lot of alien genes conferring disease and pest resistance have been transferred into wheat and mapped by *ph1b*-induced homoeologous recombination (Dong et al., 2020; Wan et al., 2020). In this study, 24 CS-*Ae*. *longissima* 6S^l^#3 recombinants were developed based on *ph1b*-induced homoeologous recombination. By integration of cytogenetic analyses and powdery mildew resistance evaluation, the novel powdery mildew resistance gene *Pm6Sl* was mapped to the distal interval of 42.8 Mb in the long arm of 6S^l^#3.

*Ae*. *longissima* has been reported to contain diverse biotic stress resistance genes. However, currently, very few genes from this species are deployed in wheat breeding programs, except *Pm13* (Ceoloni et al., 1992). In the present study, we developed two 6S^l^#3 recombinants, T27 (Ti6AS.6AL-6S^l^#3L-6AL) and R43 (T6BS.6BL-6S^l^#3L) by *ph1b*-induced homoeologous recombination. Both lines conferred broad-spectrum resistance to powdery mildew and harbored *Pm6Sl* in an *Ae. longissima* segment of <8% of the 6S^l^ genomic length. These resistant recombinants with tiny 6S^l^ segments will decrease the linkage drags and be potentially useful in wheat disease resistance breeding programs.

## Data Availability Statement

The original contributions presented in the study are included in the article/Supplementary Material, further inquiries can be directed to the corresponding author/s.

## Author Contributions

WL and HL conceived the research and wrote the paper. HL and SS performed *in situ* hybridization. XT, CM, and WM conducted full-length cDNA data analysis and molecular marker development. YZ, QC, and JM conducted molecular marker analysis. WM evaluated responses to *Bgt* isolates. JH, JQ, and ZF conducted SSR marker analyses. All authors contributed to the article and approved the submitted version.

## Funding

This research was supported by the National Natural Science Foundation of China (Nos. 31971887 and 31801361), the Scientific and Technological Research Project of Henan Province of China (No. 212102110059), and the Topnotch Talents of Henan Agricultural University (30500939).

## Conflict of Interest

The authors declare that the research was conducted in the absence of any commercial or financial relationships that could be construed as a potential conflict of interest.

## Publisher's Note

All claims expressed in this article are solely those of the authors and do not necessarily represent those of their affiliated organizations, or those of the publisher, the editors and the reviewers. Any product that may be evaluated in this article, or claim that may be made by its manufacturer, is not guaranteed or endorsed by the publisher.

## Supplementary Material

Supplementary Figure S1 Characterization of 6S^l^#3 recombinants by wheat SSR markers and 6S^l^#3-specific markers. Wheat 6A chromatins were represented by red column, 6B by purple and 6S^l^#3 by green. Wheat 6A and 6B SSR markers were in red and purple italic, respectively, ordered by locating chromosome bin and primer-aligned CS reference genomic sequences in the same bin; 6S^l^#3-specific markers in green bold, ordered by derived full-length cDNA sequence-aligned *Ae. longissima* TL05 reference genomic sequences. Bold black numbers on the right of 6A and 6B columns represented chromosome fragment length (FL). Based on analyses of SSR markers and 6S^l^#3-specific markers, T31 was designated as T6S^l^#3S.6S^l^#3L-6AL, R43 as T6BS.6BL-6S^l^#3L, T24 as T6S^l^#3S.6S^l^#3L-6AL, and T27 as Ti6AS.6AL-6S^l^#3L-6AL.

Supplementary Table S1 SSR primers used in this study.

Supplementary Table S2 Characterization of 6S^l^#3 recombinants using wheat SSR markers.
