# Supplementary figures and images for: Development and Characterization of Triticum aestivum-Aegilops longissima 6Sl Recombinants Harboring a Novel Powdery Mildew Resistance Gene Pm6Sl
Source: Front Plant Sci. 2022 Jun 2;13:918508. doi: 10.3389/fpls.2022.918508 (PMC9201914; doi:10.3389/fpls.2022.918508)

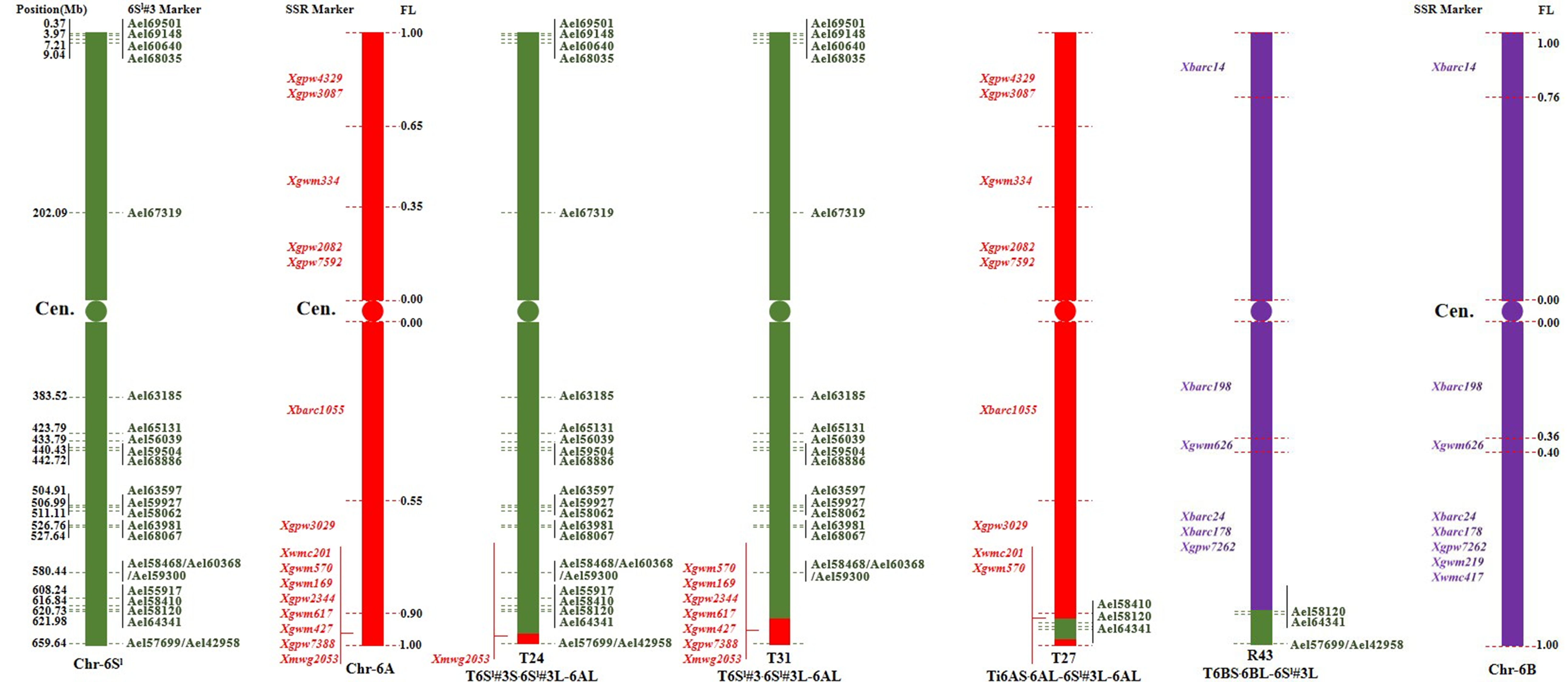

Supplement: Supplementary Figure S1 — Characterization of 6Sl#3 recombinants by wheat SSR markers and 6Sl#3-specific markers. Wheat 6A chromatins were represented by red column, 6B by purple and 6Sl#3 by green. Wheat 6A and 6B SSR markers were in red and purple italic, respectively, ordered by locating chromosome bin and primer-aligned CS reference genomic sequences in the same bin; 6Sl#3-specific markers in green bold, ordered by derived full-length cDNA sequence-aligned Ae. longissima TL05 reference genomic sequences. Bold black numbers on the right of 6A and 6B columns represented chromosome fragment length (FL). Based on analyses of SSR markers and 6Sl#3-specific markers, T31 was designated as T6Sl#3S.6Sl#3L-6AL, R43 as T6BS.6BL-6Sl#3L, T24 as T6Sl#3S.6Sl#3L-6AL, and T27 as Ti6AS.6AL-6Sl#3L-6AL. [file Image_1.TIF]
